# Southern Discomfort?: PON1 Variation May Help Explain Regional CVD Risk

**DOI:** 10.1289/ehp.117-a359b

**Published:** 2009-08

**Affiliations:** **Kris S. Freeman** has written for *Encarta* encyclopedia, NIH, ABCNews.com, and the National Park Service. Her research on the credibility of online health information appeared in the June 2009 *IEEE Transactions on Professional Communication*

Age-associated rates of cardiovascular disease (CVD) are higher in the Southern states (except Florida) than anywhere else in the United States, and higher among Southern blacks than Southern whites. Animal studies suggest that higher levels of activity of the enzyme paraoxonase-1 (PON1) may lower the risk of CVD, specifically atherosclerosis, but evidence linking functional variations in PON1 to atherosclerosis risk in humans has been ambiguous. New research correlates functional variation in PON1 activity with race, which may help explain demographic variation in CVD prevalence **[*EHP*** **117:1226–1231; Davis et al.]**.

PON1 is carried by high-density lipoprotein in the blood and is involved in the hydrolysis, or breakdown, of oxidized low-density lipoprotein, whose buildup is considered an early step in the development of atherosclerosis. PON1 is also involved in the hydrolysis of the toxic oxon metabolites of certain organophosphate insecticides.

A single-nucleotide polymorphism at position 192 on the *PON1* gene results in two functional variations of the enzyme, the Q and R forms. Other studies have associated the R form with a greater risk of atherosclerosis. The R form also hydrolyzes chlor pyrifos oxon more effectively than the Q form, whereas both alloforms are equally effective at metabolizing diazoxon; neither hydrolyzes paraoxon quickly enough to offer protection. PON1 activity is also linked to the quantity of the enzyme in the blood, which is controlled in part by polymorphisms in the gene’s promoter region and may vary by at least 13-fold among individuals.

The researchers analyzed serum samples for 200 adult black and white men and women (50 in each sex–race group) obtained from blood banks in Alabama and Tennessee. The team determined PON1 functional genotypes—RR, QR, or QQ—by measuring rates of hydrolysis of paraoxon and diazoxon. They also analyzed arylesterase activity (another measure of PON1 activity), levels of cotinine (a biomarker of smoking, which can affect PON1 levels), and C-reactive protein (a biomarker of inflammation associated with greater risk of CVD).

Forty-four percent of black subjects had higher *in vitro* rates of paraoxon hydrolysis and lower rates of diazoxon hydrolysis, consistent with the RR genotype that has been hypothesized to increase the risk of CVD. In contrast, only 7% of white subjects had activity levels consistent with the RR genotype. Black subjects also had higher levels of C-reactive protein, consistent with a greater risk of CVD. However, levels of C-reactive protein were not associated with PON1 activity. Cotinine levels indicated that all study participants were nonsmokers—possibly the result of the blood banks having screened out smokers.

The authors conclude their data support the idea that the functional RR genotype is less protective of cardiovascular health. They are working on a follow-up study of Southerners of both races and sexes where there is more information about participants’ health status and medical history.

## Figures and Tables

**Figure:**
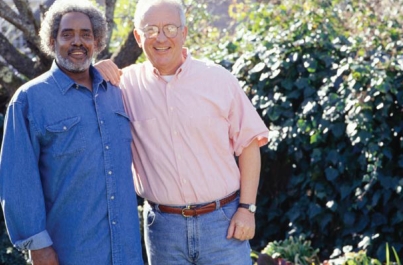
PON1 helps break down pesticides in the body.

